# Biological Activities of Molecules Derived from *Olea europaea* L. Tested *In Vitro*

**DOI:** 10.3390/life14010049

**Published:** 2023-12-28

**Authors:** Giulia Marrone, Silvia Urciuoli, Eleonora Candi, Roberta Bernini, Gianluca Vanni, Claudia Masci, Cristina Guerriero, Mara Mancini, Antonino De Lorenzo, Pamela Vignolini, Annalisa Noce

**Affiliations:** 1 Department of Systems Medicine, University of Rome Tor Vergata, 00133 Rome, Italy; 2 PHYTOLAB (Pharmaceutical, Cosmetic, Food Supplement, Technology and Analysis)—DiSIA, University of Florence, 50019 Florence, Italy; silvia.urciuoli@unifi.it (S.U.);; 3 Department of Experimental Medicine, University of Rome Tor Vergata, Via Montpellier 1, 00133 Rome, Italy; 4 Istituto Dermatopatico Dell’Immacolata—IDI, Istituto di Ricovero e Cura a Carattere Scientifico—IRCCS, Via Monti di Creta 104, 00166 Rome, Italy; 5 Department of Agriculture and Forest Sciences (DAFNE), University of Tuscia, Via San Camillo de Lellis, 01100 Viterbo, Italy; 6 Breast Unit, Department of Surgical Science, Policlinico Tor Vergata University, 00133 Rome, Italy; 7 Section of Clinical Nutrition and Nutrigenomic, Department of Biomedicine and Prevention, University of Rome Tor Vergata, 00133 Rome, Italy; 8 UOSD Nephrology and Dialysis, Policlinico Tor Vergata, 00133 Rome, Italy

**Keywords:** *Olea europaea* L., Mediterranean diet, hydroxytyrosol, oleuropein, chronic degenerative non-communicable diseases, HEK-293E, chronic kidney disease, IncuCyte

## Abstract

Background: Extra virgin olive oil is a typical food of the Mediterranean area, obtained by pressing *Olea europaea* L. fruits. Its polyphenols have been studied for their antioxidant function and protective action against cancer and chronic kidney disease. In this *in vitro* study, we tested titrated extracts from *Olea europaea* L. on a human embryonic kidney 293 (HEK-293E) cell line, regarding their pro-apoptotic and antiproliferative capacities, using “ IncuCyte^®^ S3 Live-Cell Analysis System”. Materials and Methods: We selected *Olea europaea* L. active compounds like hydroxytyrosol (HT) and oleuropein (OLE). These extracts were tested at different concentrations and characterized by HPLC-DAD-MS for the content in secondary active metabolites. The real-time observation of cell behavior was performed by IncuCyte, which can quantitatively analyze the cell proliferation and death. Results: This study showed that all the tested extracts can significantly inhibit cellular growth at 50 µM but the reduced proliferation is not related to an increase in cellular apoptosis. Instead, the same analysis performed by using extracts at 100 µM reveals that they can inhibit cellular growth, further inducing cellular apoptosis. Conclusions: The results on the HEK-293E cells confirmed the antiproliferative and proapoptotic actions of active compounds from an *Olea europaea* L. matrix in this cell line.

## 1. Introduction

The Mediterranean diet (MD) is considered a useful tool in the prevention and treatment of chronic degenerative non-communicable diseases (CDNCDs). In particular, the MD plays a key role in the clinical management of neoplastic and dysmetabolic pathologies, characterized by a chronic low-grade inflammatory state [1]. MD can counteract the incidence of cancer, given its protective effects in reducing cellular oxidative and inflammatory processes and in avoiding DNA damage, cell proliferation, angiogenesis and metastasis [2].

Various studies have demonstrated that MD provides high levels of phytochemicals, including polyphenols, which seem to exert beneficial biological effects, including antioxidant, anti-inflammatory, immunomodulatory, antitumoral and antidiabetic activities [2,3,4,5,6].

Extra virgin olive oil (EVOO) represents the main source of polyunsaturated fatty acids in MD, and it belongs to the plant world [7]. The consumption of EVOO has been largely associated with numerous health benefits. In fact, the European Food Safety Authority (EFSA) approved the EVOO claim, as defined in Commission Regulation (EU) no. 432/2012 [8].

The main clinical trial that demonstrated the anticancer properties of EVOO was the European Prospective Investigation into Cancer and Nutrition (EPIC) study that highlighted the relationship between cancer and nutrition, taking into account different factors that impact lifestyle (nutritional habits, medical history, anthropometric measurements, nutritional status and laboratory parameters). The authors concluded that the MD is a valuable nutritional model to prevent cancer [9,10]. The Italian cohort of the EPIC study analyzed the impact of dietary habits on cancer incidence. This study demonstrated that the subjects that followed the food pattern “Olive oil & Salad” were related to a lower mortality and a lower risk of cancer onset, compared to the food pattern “Pasta & Meat” [11].

The scientific literature indicates that EVOO beneficial effects are ascribed, at least in part, to the presence of some phenolic compounds, which are well recognized for their remarkable antioxidant and anticancer activities [12]. Among these, the most representative are oleuropein (OLE), hydroxytyrosol (HT) and oleocanthal. The first one is the main glycoside in olives, and its degradation induces the formation of HT in olive oil [13]. These polyphenols are present not only in the fruit but also in the olive leaves. In fact, olive leaves, usually considered a waste in the olive oil sector, are an important source of polyphenols. The main natural bioactive compound (NBC) contained in olive leaves is the OLE from, which HT derives [14].

Therefore, the NBC can also be obtained through circular economy models that promote green technologies, which allow for NBC recovery from by-products and wastes. In fact, currently, a model of circular economy is already operating in the olive oil sector. In this regard, an industrial platform to produce micronized powders and extracts of *Olea europaea* L. was described by Romani et al., using advanced drying techniques, membrane technologies and evaporation [15]. This platform is eco-sustainable and allows one to obtain final products, using water as an extraction solvent and, thus, avoiding toxic solvents. Micronized powders and extracts obtained via this platform are standardized in terms of polyphenol content [16]. This platform was used in this *in vitro* study in order to obtain the extracts to test.

**Figure 1:**
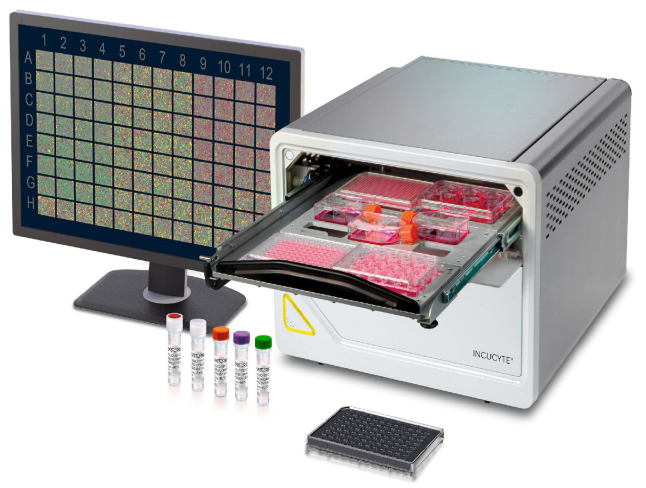
IncuCyte S3 Live-Cell Analysis System.

The purpose of this *in vitro* study is to test the NBCs from *Olea europaea* L., such as HT standard, OLE standard and two characterized extracts from olive leaves on human embryonic kidney 293 (HEK-293E) cells, regarding their potential pro-apoptotic and antiproliferative capacities, using the “IncuCyte^®^ S3 Live-Cell Analysis System” (Sartorius) (Figure 1). The latter is a live-cell imaging technology, and it permits real-time visualization, the characterization and the measurement of biological processes in living cells by using time-lapse microscopy. In fact, the IncuCyte^®^ S3 Live-Cell Analysis System is useful for the evaluation of cell health, such as proliferation, cytotoxicity and apoptosis. The advantages of IncuCyte^®^ S3 are easy sample preparation, image acquisition for selected periods of time, automatic settings of the cell parameters to be monitored and real-time visualization. This technology will play an important role in the development and validation of the next generation of cancer immunotherapies.

## 2. Materials and Methods

### 2.1. Extracts Tested In Vitro

In this study, the active molecules of *Olea europaea* L. were tested [14]. We selected a commercial standard of 98% HT (Merck, Darmstadt, Germany), a commercial standard of 98% OLE (Merck, Darmstadt, Germany) and two olive leaves extracts obtained through circular recovery of wastes from the EVOO production chain. For the chemical characterization of the olive leaves extracts, we used the HPLC-DAD-MS HP-1260 liquid chromatograph Infinity II, equipped with a DAD detector and an LC/MSD API electrospray (Agilent Technologies, Santa Clara, CA, USA), operating in negative and positive ion mode. The mass spectrometer operating conditions were as follows: gas temperature 350 °C at a flow rate of 10.0 µL/min, nebulizer pressure 30 psi, quadrupole temperature 30 °C and capillary voltage 3500 V. The fragmentor was set at 120 eV. Polyphenols were analyzed by using a column Lichrosorb RP18 250 × 4.60 mm i.d, 5 µM (Merck, Darmstadt, Germany). The eluents were H_2_O adjusted to pH 3.2 with HCOOH and CH_3_CN. A four-step linear solvent gradient was used, starting from 100% H_2_O up to 100% CH_3_CN, for 117 min at a flow rate of 0.8 mL/min. The chromatograms were acquired at 280 nm and 330 nm. The quantification of polyphenols was determined by using external calibration curves. HT, tyrosol and OLE were quantified at 280 nm, and calibration curves with r^2^ ≥ 0.9998 were considered. The concentrations of the individual compounds were calculated by applying the appropriate corrections for changes in molecular weight.

### 2.2. In Vitro Study

To examine the effects on cell proliferation and caspase 3/7 activation on the HEK-293E cells, phenolic compounds with known functional and biological activities were selected from the *Olea europaea* L. matrix, such as HT and OLE, both present in the olive leaves. The selected natural extracts and standards were tested at different concentrations (50 and 100 µM). The real-time observation of cell behavior was made possible thanks to the use of IncuCyte^®^ S3 Live-Cell Analysis System (Sartorius, Göttingen, Germany), which can quantitatively analyze cellular proliferation and death. These parameters were monitored using a fluorescent dye reagent, specific for live cell nuclei and non-toxic, called “NucLight Rapid Red Reagent (Sartorius, cat.n. 4717). This reagent is a DNA cell-permeable dye that specifically stains nuclei in live cells, allowing for the real-time quantification of cell growing. In addition, apoptosis was quantified and was analyzed using the IncuCyte Caspase-3/7 reagent Green, able to bind the activated caspases 3/7 and to emit a fluorescent signal (Sartorius, cat.n. 4440). The molecule couples the activated caspase-3/7 recognition motif (DEVD) to a DNA intercalating dye, and it is ideal for the real-time quantification of cells undergoing caspase-3/7-mediated apoptosis. Furthermore, it is formulated specifically for IncuCyte^®^ S3 Live-Cell Analysis System and it can be added directly to culture cells to acquire live cell images [17]. In particular, cell growth and apoptosis were monitored, respectively, every 2 and 6 h, for 36 h, using IncuCyte^®^ S3 Live-Cell Analysis System (Sartorius), and the related software was used to make the growth curve and the objective counts.

Statistical analyses were performed using GraphPad PRISM 9.3.0 software (GraphPad Software, San Diego, CA, USA). The significance was calculated with Student’s *t*-test * *p* < 0.05; ** *p* < 0.001.

## 3. Results

**Figure 2:**
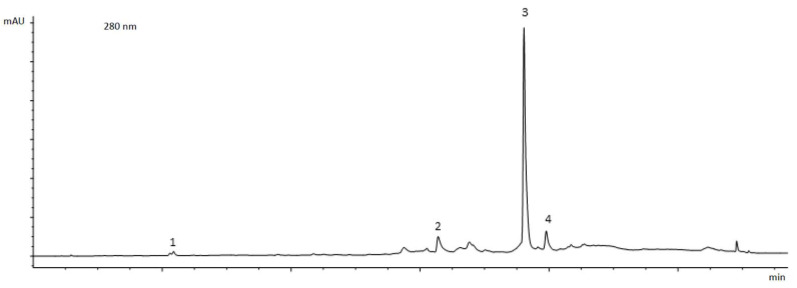
Chromatographic profile of olive leaves extract (Olea extract 20) at 280 nm. 1. hydroxytyrosol; 2. verbascoside; 3. oleuropein; 4. secoiridoid derivative.

In Figure 2, we show a chromatographic profile, acquired at 280 nm, of one of the tested extracts (Olea extract 20).

HPLC-DAD-MS analysis of the olive leaves extracts showed that the OLE concentration for the first extract was 20% (Olea extract 20) and for the second extract was 40% (Olea extract 40). The other compounds present in the extracts are verbascoside and secoiridoid derivatives.

The results were obtained by using the IncuCyte^®^ S3 software analyzer, version number V2018B (Sartorius, Germany).

The human kidney cell lines in culture, for the *in vitro* study, were assessed using the IncuCyte^®^ S3 Live-Cell Analysis System in order to evaluate the proapoptotic and antiproliferative activities of olive leaves extracts. We conducted the experiments with the standards and with the extracts at concentrations of 50 and 100 µM.

**Figure 3:**
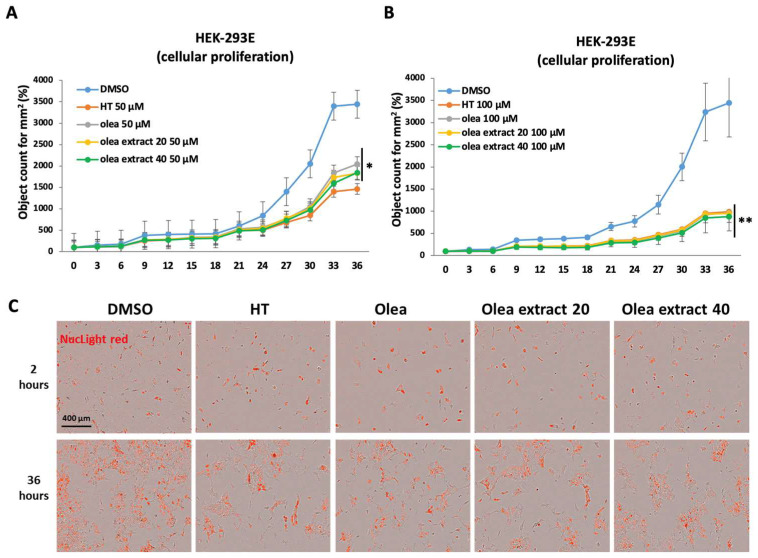
Evaluation of antiproliferative activity of OH-ty (HT), Oleuropein (Olea), Olea extract 20% (Olea extract 20) and Olea extract 40% (Olea extract 40) on the HEK-293E cell line. Panel (**A**): antiproliferative action of 50 µM HT, Olea, Olea extract 20 and Olea extract 40, compared with control (DMSO). Panel (**B**): antiproliferative action of 100 µM HT, Olea, Olea extract 20 and Olea extract 40, compared with control (DMSO). Panel (**C**): representative images of treated cells and stained with NucLight Red reagent. Test *t* Student * *p* < 0.05; ** *p* < 0.001.

This *in vitro* study highlighted that the olive leaves extracts are able to significantly inhibit the cellular growth of the HEK-293E cells both at 50 and 100 µM after 36 h of treatment, when compared to the control (DMSO) (Figure 3).

**Figure 4:**
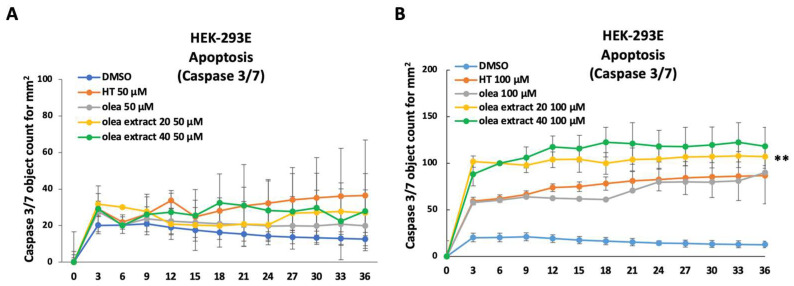
Evaluation of proapoptotic activity of OH-ty (HT), Oleuropein (Olea), Olea extract 20% (Olea extract 20) and Olea extract 40% (Olea extract 40) on the HEK-293E cell line. Panel (**A**): caspase 3/7 activation in cells treated with 50 µM HT, Olea, Olea extract 20 and Olea extract 40, compared with control (DMSO). Panel (**B**): caspase 3/7 activation in cells treated with 100 µM HT, Olea, Olea extract 20 and Olea extract 40, compared with control (DMSO). Test *t* Student ** *p* < 0.001.

Regarding cellular apoptosis, we observed that the cell treatment with the olive leaves extracts at 50 µM had no effect on cellular apoptosis (Figure 4A), while at 100 µM, the treatment could significantly induce cellular apoptosis, as shown by caspase 3/7 activation (Figure 4B).

Altogether, our results confirm the antiproliferative effect of the HT standard, OLE standard and the two characterized extracts (Olea extract 20 and Olea extract 40) obtained from olive leaves on human embryonic kidney 293 (HEK-293E) cells. In addition to the antiproliferative action, the tested molecules also exerted a proapoptotic effect on HEK-293E, at a concentration of 100 µM.

## 4. Discussion

Several epidemiological and preclinical studies highlighted the beneficial effects of EVOO in cancer prevention, mainly related to its antioxidant power [14,18,19,20,21]. In fact, EVOO is characterized by healthy proprieties, such as cardioprotective, antilipidemic and anti-inflammatory, and its consumption is considered an adjuvant therapy in the clinical management of CDNCDs [8,22,23,24,25]. The most beneficial compounds present in EVOO are polyphenols, namely secondary plant metabolites, which have been studied extensively for their health-promoting properties. These compounds also exert antineoplastic activities, like cytotoxic activity against cancer cells and anti-inflammatory effects, that are considered a promoting factor of carcinogenesis starting from the initial stages.

Following an MD, consuming EVOO as the principal source of fats, seems to be correlated with a reduced overall risk of cancer, particularly for the gastroenteric system, prostate and breast [2,14,23,26,27]. The antineoplastic activities of EVOO, as well as of its specific fractions or isolated compounds, have been widely studied and evidenced both in *in vitro* study on cell cultures and in in vivo study (in animal models and in clinical trials) [28,29,30,31,32].

These EVOO antineoplastic activities seem to be mediated by natural bioactive compounds, such as tocopherols, β-carotene and minor polar compounds (MPCs) [7]. In this regard, several researches have demonstrated that olive oil decreases the incidence of cancer [33,34]. In particular, in colorectal cancer, HT and OLE seem to inhibit the transformation of normal ileal and colon mucosa into neoplasia [35]. A further study demonstrated, in an animal model, the antineoplastic effect of olive oil against colon carcinogenesis; in fact, HT and OLE are able to counteract COX-2 over-expression. This altered enzyme activity seems to be related to colorectal neoplasia, as it promotes cell growth, angiogenesis and Bcl-2 expression [36,37]. The down-regulation of COX-2 in colorectal cancer, induced by OLE, appears to be linked to the down-regulation of the wnt/-catenin pathway [38]. In fact, several studies demonstrated the hyperactivation of wnt/-catenin in some types of cancer, such as gastric, colorectal and endometrial [38,39,40]. Morana et al. [41] pointed out that the beneficial effects on cancer induced by OLE are related to its concentration, to the time of exposure and to the kind of cancer. Moreover, the antiproliferative effect of OLE has been highlighted in both *in vitro* and in vivo models. In detail, Bossio et al. [42], in an *in vitro* study, showed that OLE induces antiproliferative action on seminoma cell lines (namely TCAM-2 and SEM-1) through the inhibition of NF-κB. In an animal breast cancer model, Ci et al. [43] demonstrated that the administration of OLE at a dose of 125 mg/kg was able to reduce peri pulmonary and parenchymal lung metastasis. OLE also seems to be effective in the treatment of prostate cancer. In fact, in an *in vitro* study, Papachristodoulou et al. [44] demonstrated that doxorubicin (DXR) and OLE are able to inhibit PC-3 cell proliferation and to induce autophagy. Moreover, the combined treatment (DXR and OLE) causes a more powerful cellular inhibition compared to the single treatment, reducing the possible side effects. In fact, DXR can induce cardiotoxicity, and the combined treatment DXR plus OLE seems to prevent cardiomyopathy [45]. Therefore, OLE and its metabolite HT seem to exert not only a chemopreventive function but also empower the effectiveness of chemotherapeutic drugs, permitting their use at lower dosages, thus reducing the possible side effects [13,34,46]. Another study that emphasized the adjuvant chemotherapeutic role of OLE was conducted by Ruzzolini et al. on A375 human melanoma cells, showing that OLE at a concentration of 500 μM stimulates apoptosis and that, at lower dosages (namely 250 μM), is able to interfere in cell proliferation and in the impairment of the pAKT/pS6 pathway. Therefore, these interesting results confirm the potential additive role of OLE to traditional chemotherapeutic agents against melanoma cells [47].

Other factors related to carcinogenesis are reactive oxygen species (ROS) and nitrogen species (NOS), and, although they are essential for cell functions, if overexpressed, can induce DNA, lipid and protein damage [48]. There is a unanimous consensus that olive leaves phenolics have a strong ability to scavenge NOS and to quench ROS production [49]. In particular, OLE is able to chelate copper and iron metal ions that are involved in the formation of free radicals [50]. A further *in vitro* study conducted on a human colorectal cancer cell line LS180 demonstrated that HT is able to induce apoptosis, enhancing *CASP3* gene expression and the *BAX:BCL2* ratio. Moreover, HT increased the activity of several antioxidant enzymes, such as catalase, superoxide dismutase and glutathione peroxidase, thus counteracting ROS production [51]. In both *in vitro* and in vivo studies, that examined the possible effects of olive leaves on inflammation and on cancer cells, it has been shown that olive leaves polyphenols exert anti-inflammatory and protective activities against DNA damage caused by free radicals [52,53,54]. These bioactive properties of olive leaves polyphenols could explain the preventive action and the slow-down of cancer progression induced by EVOO NBCs [55]. In detail, EVOO polyphenols are able to counteract inflammation, involving the nuclear factor kappa-light-chain enhancer of activated B cell (NF-κB) pathway [56]. Specifically, the activation of downstream effectors of NF-κB, such as interleukin (IL)-12, tumor necrosis factor-related apoptosis-inducing ligand (TRAIL), interferon (IFN)-γ and transcription factors, can engage antitumor immunity [57]. These protective factors, if produced at lower concentrations, can trigger an impaired micro-environment that facilitates the development or the progression of cancer [58,59].

In our *in vitro* study, the results obtained on the HEK-293E cells confirmed the antiproliferative and proapoptotic actions of phenolic compounds of *Olea europaea* L. on this cell line, confirming the potential beneficial action of MPCs in the clinical management of CDNCDs. The results obtained at 100 uM show that both extracts (Olea extract 20 and Olea extract 40) have antiproliferative and proapoptotic actions on HEK-293E cells, comparable to that of the standards (HT and Olea). These results suggest a greater activity of the phytocomplex of natural extracts from olive leaves, compared with *Olea europaea* L. standards at the same concentration. These data allow us to speculate on the synergic action of the phytocomplex.

A further novelty of this study is that we used the next-generation IncuCyte^®^ S3 Live-Cell Analysis System that automatically acquires and analyzes images, without removing cell lines from the incubator. To date, there are no reports in the literature that have evaluated the anti-proliferative effects of OLE and its metabolites using a real-time live cell system. In fact, this instrument is able to evaluate cell viability by placing a microscope inside the incubator, permitting one to detect live cell images in a deeper and more physiological way, allowing one to obtain relevant information about the cell status. Further, real-time live cell monitoring using a fluorescent non-toxic dye reagent, specific for live cell nuclei and the activated caspases 3/7, enables the evaluation and monitoring of cell behavior, growth and cellular apoptosis activation in real time and not at an endpoint, without altering the cell culture condition. This point is crucial to avoid artifacts of the treatment that can influence the final results.

A future field of application for IncuCyte should be represented by the monitoring of chemotherapeutic efficacy in cancer patients.

In light of these results, we believe that micronized powders and extracts, obtained with circular economy models, could be used for the production of natural food supplements as adjuvant therapy in cancer patients. In this regard, these encouraging results obtained *in vitro* should be confirmed in in vivo studies in animal models and then in randomized clinical trials.

Several studies highlighted that EVOO polyphenols seem to represent a new adjuvant therapy in counteracting the carcinogenic process. For this reason, it is important to encourage the market production of polyphenols, also using circular economy models, economically sustainable and aimed at a reduction in environmental impacts [15,60,61,62]. Therefore, the use of extracts derived from EVOO by-products, obtained through models of the circular economy, can represent a new potential strategy to prevent and treat CDNCDs, in particular, cancer.

## 5. Conclusions

Our results on the HEK-293E cells confirm the antiproliferative and proapoptotic actions of HT and OLE from an *Olea europaea* L. matrix, in this cell line. Moreover, our study emphasizes the relevant role of the IncuCyte^®^ S3 Live-Cell Analysis System in the field of molecular biology, in the polyphenols field, allowing us to monitor their effects in a time-dependent manner, using live cell imaging.

## Data Availability

Data are contained within the article.
